# Phase and absorbance retrieval in X-ray holographic microscopy under weak illumination using physics-driven neural networks

**DOI:** 10.1107/S1600577526003188

**Published:** 2026-04-10

**Authors:** Jihwan Kim, Jun Lim, Sugeun Jo, Sungho Park, Sang Joon Lee

**Affiliations:** a Department of Mechanical Engineering Pohang University of Science and Technology Pohang Republic of Korea; b Pohang Accelerator Laboratory Pohang University of Science and Technology Pohang Republic of Korea; c Department of Radiology, Children’s Hospital Colorado, University of Colorado, Anschutz Medical Campus, Aurora, CO, USA; Tohoku University, Japan

**Keywords:** X-ray holographic microscopy, single-shot reconstruction, physics-driven neural network, neural field

## Abstract

X-ray holographic microscopy is a three-dimensional (3D) imaging technique for nanoscale-resolution imaging of morphological features and phase contrast in biological samples and solid-state materials. However, it is challenging to recover phase and absorbance information from shot-noise-limited (SNL) X-ray holograms acquired under weak illumination. In this study, we propose a deep learning model, named MorpHoloNet-X, for single-shot phase and absorbance retrieval from SNL X-ray holograms using a physics-driven neural network. By incorporating physics-based prior knowledge and wave propagation principles into the neural network, MorpHoloNet-X can directly reconstruct 3D complex wavefield, phase, and absorbance distributions in a simulated 3D volume. The performance of the proposed MorpHoloNet-X is validated using synthetic and experimental SNL holograms, and the results are compared with those of conventional methods. The proposed technique would be utilized to reconstruct phase and absorbance information from hard X-ray holograms acquired under rapid acquisition or weak illumination.

## Abstract 2

A physics-driven deep learning model enables single-shot retrieval of 3D phase and absorbance from shot-noise-limited hard X-ray holograms. Validated on both synthetic and experimental data, it provides robust reconstruction under weak illumination.

## 1. Introduction

X-ray holographic microscopy (XHM), originating from Gabor’s holography (Gabor, 1948 ▸), is a nondestructive three-dimensional (3D) imaging technique for visualizing internal structures of test samples (Aoki & Kikuta, 1974 ▸; Jacobsen *et al.*, 1990 ▸; McNulty *et al.*, 1992 ▸; Faigel & Tegze, 1999 ▸). Holographic patterns are generated by interference between a coherent incident beam and the object wave scattered by test samples at the detector plane. For phase-contrast imaging, a quasi-monochromatic X-ray beam has been utilized to analyze the 3D structures of animal brains and lungs under *in situ* conditions (Kitchen *et al.*, 2005 ▸, Croton *et al.*, 2018 ▸). To better resolve submicrometer features, a Fresnel zone plate is commonly employed to focus a soft X-ray beam onto a pinhole, generating a divergent cone beam with increased spatial coherence (Heine *et al.*, 2011 ▸; Lim *et al.*, 2011 ▸). The cone-beam illumination is then used to acquire holographic signals of biological cells, carbon powders, and gold patterns. For hard X-ray applications, a nanoscale pinhole needs to be fabricated in a metal substrate thick enough to block X-ray transmission in regions outside the pinhole area. However, fabricating high-aspect-ratio pinholes by mechanical machining or chemical etching is technically challenging.

Based on keyhole coherent diffractive imaging (Abbey *et al.*, 2008 ▸), two orthogonally aligned Kirkpatrick–Baez (KB) mirrors are used to focus an incident hard X-ray beam into a submicrometer focal spot (Kuan *et al.*, 2020 ▸; Kalbfleisch *et al.*, 2022 ▸). The KB mirrors form a divergent hard X-ray cone beam without the need for a pinhole, enabling high-resolution 3D tomographic analysis of a metal test pattern, a chalk sample, and neuronal morphologies in *Drosophila*. To further enhance the resolution of XHM, the hard X-ray beam focused by KB mirrors passes through an X-ray waveguide to reduce the focal spot size (Bartels *et al.*, 2015 ▸; Soltau *et al.*, 2021 ▸). The X-ray waveguide channel is usually fabricated by e-beam lithography, etching, wafer bonding, and wafer dicing (Neubauer *et al.*, 2014 ▸). The resolution of XHM can be improved as the effective diameter of the X-ray waveguide channel decreases.

The 7C X-ray Nano Imaging (XNI) beamline at Pohang Light Source-II (PLS-II) is a third-generation synchrotron beamline that provides X-ray nanotomography and X-ray absorption near-edge structure (XANES) imaging for nano­scale-resolution analysis of internal sample structures (Lim *et al.*, 2013 ▸; Lim *et al.*, 2014 ▸; Jo *et al.*, 2025 ▸). At the 7C XNI beamline, a beryllium parabolic compound refractive lens (CRL) is used to focus hard X-rays into a small focal spot of 70 µm × 10 µm. The photon flux density in the focal spot is approximately 10^9^ photons µm^−2^ s^−1^. To implement XHM at the 7C XNI beamline by following Salditt’s work (Soltau *et al.*, 2021 ▸), the hard X-ray beam focused by the CRL passes through a 140 nm × 120 nm waveguide channel. Considering the diameter of the cone-beam illumination recorded on the detector plane, the mean photon flux per pixel is evaluated to be approximately 60 photons pixel^−1^ s^−1^. This value is markedly lower than the photon flux obtained with KB mirrors. Therefore, holographic images recorded with the hard X-ray beam at low photon flux density are shot-noise dominated and exhibit a low signal-to-noise ratio (SNR).

Conventional iterative phase retrieval methods based on the Gerchberg–Saxton (GS) algorithm have achieved high phase reconstruction accuracy with robust convergence for noise-free holograms (Gerchberg & Saxton, 1972 ▸; Latychevskaia & Fink, 2007 ▸, 2015 ▸; Latychevskaia, 2019 ▸). However, its convergence performance degrades due to the presence of noise, leading to large errors in the recovered phase and absorbance information. In particular, shot noise has been reported to induce phase fluctuations during holographic reconstruction (Charrière *et al.*, 2006 ▸; Charrière *et al.*, 2007 ▸; Lesaffre *et al.*, 2012 ▸). To achieve high magnification in XHM, the waveguide–object distance is typically set to be much shorter than the object–detector distance. Consequently, over the long propagation distance, noise-induced spurious holographic signals are accumulated during hologram reconstruction, leading to large measurement errors.

Shot noise is not a dominant limitation in many holographic imaging experiments. For visible-light holographic microscopy, shot-noise effects can be suppressed by simply increasing the illumination power without exceeding specimen-safe limits. In XHM, the photon flux density of the focused beam is typically high enough to ensure a high SNR. Meanwhile, dose-limited imaging modalities, such as low-dose cryogenic electron microscopy that minimizes radiation damage to biological samples (Glaeser, 2013 ▸; Cheung *et al.*, 2020 ▸) and high-speed holographic imaging for volumetric tracking in flow cytometry (Zhang *et al.*, 2023 ▸), require dedicated reconstruction strategies to be adopted under severe shot-noise conditions. Therefore, to overcome the hardware limitations at the 7C XNI beamline, it is required to develop a phase and absorbance retrieval method under weak illumination conditions.

Several phase retrieval methods have been introduced for holograms with shot noise. Early analytical approaches based on first Born and Rytov approximations were utilized to derive explicit solutions for phase and amplitude retrieval from in-line X-ray images (Gureyev *et al.*, 2004 ▸). Although these approaches were reported to be sensitive to shot noise due to low image contrast, experimental results showed that the maximum phase shift could be recovered with reasonable accuracy. The iterative Gabor averaging algorithm was developed by integrating iterative the GS phase retrieval method for low-frequency components with the Gabor averaging method for high-frequency components in order to reduce phase fluctuations (Rogalski *et al.*, 2025 ▸). This approach requires multiple in-line holograms acquired at different depths and illumination wavelengths. With the aid of artificial intelligence (AI), various neural-network-based methodologies have been introduced for phase retrieval (Wang *et al.*, 2024 ▸). Supervised learning approaches using mixed-scale dense convolutional neural networks (CNNs) were proposed for simultaneous retrieval of phase shift and attenuation from X-ray in-line phase contrast images (Mom *et al.*, 2022 ▸). The deep Gauss–Newton algorithm unrolled a Gauss–Newton optimization scheme and combined CNNs with the imaging physics given by the forward operator and its Fréchet derivative, enabling single-distance phase and absorption retrieval (Mom *et al.*, 2023 ▸). Residual U-net (ResUNet) models with skip connections were employed for robust phase retrieval at low photon counts (Deng *et al.*, 2020 ▸; Kang *et al.*, 2020 ▸). Since holographic datasets are essential to train a ResUNet, it is challenging to obtain sufficient X-ray holograms for different types of specimens within a limited chance of beam time.

In contrast, physics-driven approaches enable single-shot phase retrieval directly from a single input hologram without the need for training datasets (Wang *et al.*, 2020 ▸; Zhang *et al.*, 2021 ▸). The phase map predicted at the object plane is forward-propagated to the detector plane. The loss between the simulated intensity map and the input hologram is then minimized to train a neural network. A self-supervised physics-driven generative adversarial network was developed to acquire phase and absorbance information from a single X-ray hologram (Yang *et al.*, 2025 ▸). Compared with the transport-of-intensity equation (Teague, 1983 ▸), the proposed approach recovers more detailed phase information up to high-frequency components. In addition, it maintains robust phase retrieval performance for noisy X-ray holograms. Meanwhile, the convergence of neural networks tends to be less stable for absorbance than for phase. Therefore, it is necessary to develop neural network architectures that can accurately reconstruct both phase and absorbance information and to conduct quantitative evaluations.

In our previous study, a physics-driven neural network named MorpHoloNet was developed for single-shot reconstruction of 3D morphology of biological cells in digital in-line holographic microscopy (Kim *et al.*, 2025 ▸). By adopting the concept of neural fields (Xie *et al.*, 2022 ▸; Liu *et al.*, 2022 ▸), MorpHoloNet predicted 3D phase shift values inside a 3D discretized volume. A coherent laser beam propagated through the 3D volume to simulate a hologram at the detector plane. MorpHoloNet was then optimized by minimizing the loss between the simulated and input holograms. It was utilized for analyzing spatiotemporal changes in 3D position, orientation, and morphology of biological cells. While the absorption of biological cells was neglected in the previous study, quantitative characterization of energy-dependent X-ray absorption is essential in XHM. Thus, the model architecture of MorpHoloNet needs to be modified so that its output layer produces phase and absorbance information.

In this study, an AI-based XHM technique is proposed for single-shot phase and absorbance retrieval under weak illumination, using a physics-driven neural network named MorpHoloNet-X. MorpHoloNet-X predicts 3D phase shift and absorbance distributions in the region near the object plane. A coherent X-ray beam is then forward-propagated through the predicted 3D space using a physics-based forward model. The loss between the simulated and input holograms at the detector plane is minimized to update model parameters of MorpHoloNet-X. Single-shot phase and absorbance retrieval with MorpHoloNet-X is demonstrated using synthetic holograms of absorbing and phase-shifting objects and experimental X-ray holograms of a resolution target. The reconstruction performance of MorpHoloNet-X is quantitatively compared with that of the GS algorithm and a typical ResUNet model. The proposed AI-based XHM technique would be utilized to accurately reconstruct the phase and absorbance information of specimens under limited photon-flux-density conditions.

## 2. Results

### 2.1. MorpHoloNet-X workflow

**Figure 1:**
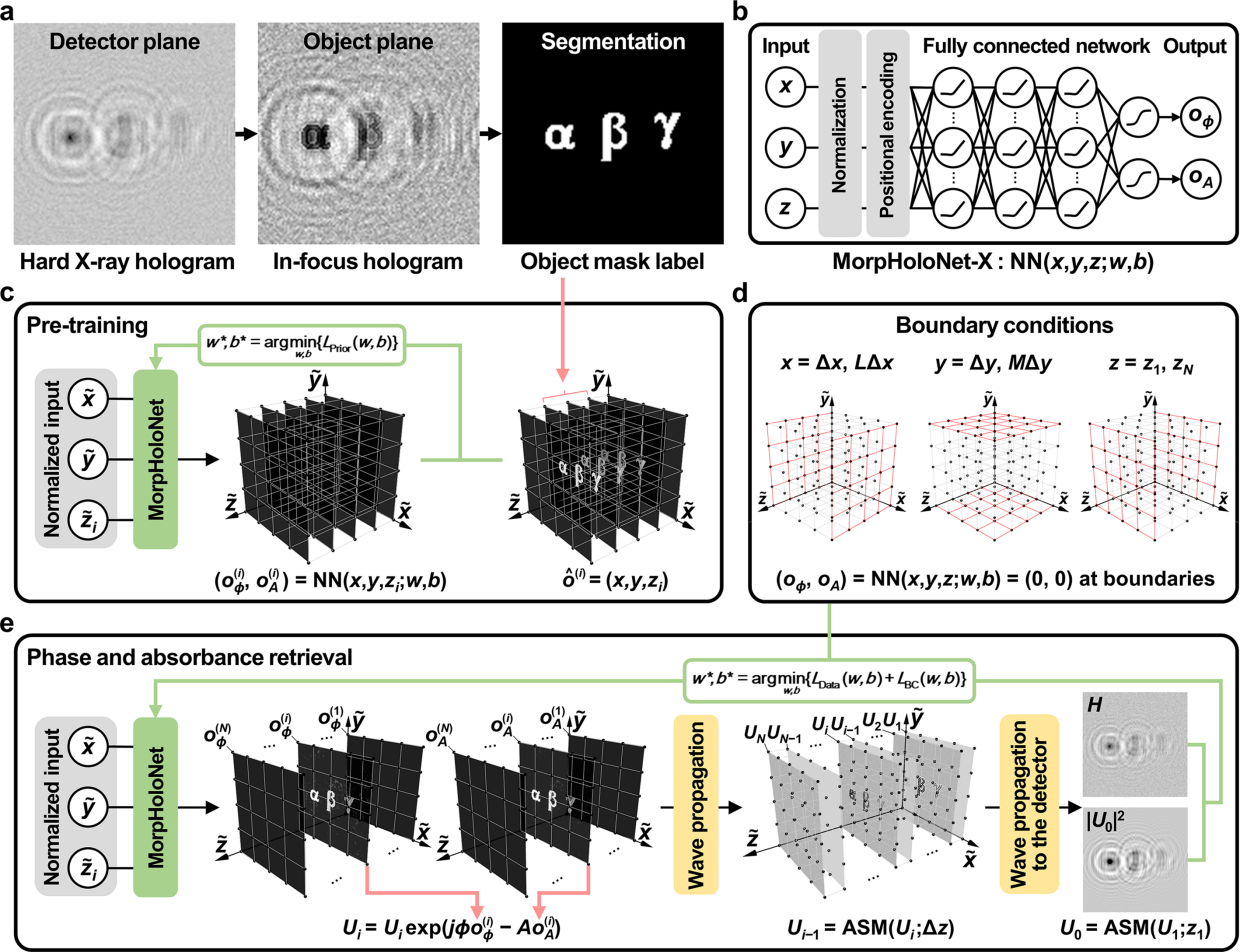
Overall workflow of the proposed MorpHoloNet-X for single-shot phase and absorbance retrieval in X-ray holographic microscopy. (*a*) Object mask generation from a hard X-ray hologram. (*b*) Neural network architecture of MorpHoloNet-X. (*c*) Pre-training process of MorpHoloNet-X using prior knowledge about object mask label arrays around the approximate depth of target objects. Training MorpHoloNet-X with (*d*) boundary conditions and (*e*) forward wave propagation using the angular spectrum method (ASM) operator.

Fig. 1 ▸ shows the workflow of MorpHoloNet-X for single-shot phase and absorbance retrieval in XHM. Using the angular spectrum method (ASM) (Goodman, 2005 ▸; Choi *et al.*, 2012 ▸), the hard X-ray hologram at the detector plane is back-propagated from the detector plane to the object plane [Fig. 1 ▸(*a*)]. From the in-focus reconstructed hologram, the two-dimensional (2D) shapes of target objects are roughly segmented to get an object mask label (see *Method* §S1 and Fig. S1(*a*) of the supporting information). In the object mask label, target object pixels are set to 1, whereas background pixels are set to 0. Discretized 3D (*x*, *y*, *z*) coordinate values are fed into MorpHoloNet-X to predict two object values (*o*) at each voxel [Fig. 1 ▸(*b*)]. The dimensions along the *x*, *y*, and *z* axes of each voxel are defined as Δ*x*, Δ*y*, and Δ*z*, respectively. Here, Δ*x* and Δ*y* are equal to the magnified pixel size of input hologram *H* at *z*_0_ = 0 (*L* × *M* pixels). *o*_ϕ_ and *o*_*A*_ denote the object values for phase and absorbance, respectively. The object values indicate whether each voxel is occupied by a medium (*o* = 0) or an object (*o* = 1). Sigmoid activation functions are employed at the output layer of MorpHoloNet-X to produce the probability of object presence at each voxel.

MorpHoloNet-X is first pre-trained with prior knowledge, including approximate depth (*z*′) values of target objects and a set of object mask label arrays around *z*′ (*z* ∈ [*z*′ − *w_z_*Δ*z*, *z*′ + *w_z_*Δ*z*]) [Fig. 1 ▸(*c*)]. The depth *z*′ can be estimated from the optical setup of XHM. *w*_*z*_ denotes the axial half-window that is adaptively chosen to be between 5 and 10. The corresponding number of the object mask label arrays is 2*w*_*z*_ + 1. Therefore, a candidate 3D region in which the target objects may exist is defined based on the multiple label arrays. Object value labels in regions far from *z*′ are constrained to 0. Both *o*_ϕ_ and *o*_*A*_ are then trained to match the corresponding label array at each depth using a mean square error (MSE) loss. The main purpose of the pre-training stage is to guide the model toward the candidate 3D region, thereby enabling robust reconstruction of the actual object region.

After pre-training, MorpHoloNet is fine-tuned with a physics-based forward model that propagates a coherent X-ray beam through 3D phase shift and absorbance distributions. The discretized depths for training MorpHoloNet-X are set to *z* ∈ [*z*′ − 40Δ*z*, *z*′ + 40Δ*z*] with intervals of Δ*z*. Here, *z*_1_ and *z*_*N*_ are equal to *z*′ − 40Δ*z* and *z*′ + 40Δ*z*, respectively. The corresponding number *N* of *z* slices is 81. On the six faces of the 3D volume (*x*_BC_ = Δ*x*, *L*Δ*x*; *y*_BC_ = Δ*y*, *M*Δ*y*; *z*_BC_ = *z*_1_, *z*_*N*_), the object values for boundary conditions (*o*_BC_) are set to 0 [Fig. 1 ▸(*d*)].

Inside the 3D volume, the complex X-ray wavefield (*U*) is propagated from *z*_*N*_ to *z*_1_ with intervals of Δ*z* based on a physics-based forward model. Since X-ray holograms are normalized by the background hologram recorded without any specimen, the incident X-ray beam *U*_*N*_ at *z*_*N*_ is defined *U*_*N*_ = 1. The complex refractive index (*n*) in each voxel is expressed as follows,
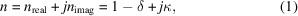
where *n*_real_ and *n*_imag_ are the real and imaginary parts of *n*, respectively. δ and κ denote the refractive index decrement and the extinction coefficient, respectively. For a voxel fully occupied by an object (*o* = 1), the corresponding phase shift (ϕ) and absorbance (*A*) coefficients are defined as follows,
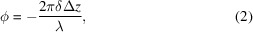

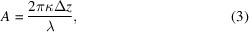
where λ is the wavelength of the X-ray beam. ϕ and *A* can be roughly initialized using refractive index values of target objects reported in other literature. The tensors representing ϕ and *A* can be optionally configured to be trainable or kept fixed.

The ASM for plane waves is employed to simulate the wave propagation between two adjacent depth planes. The propagation of the complex X-ray wavefield can be expressed as follows,
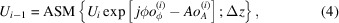
where *U*_*i*_ and *U*_*i*−1_ are the wavefield arrays at *z*_*i*_ and *z*_*i*−1_ (*i* = 2, 3,…, *N*), respectively. ASM{·} denotes the wave propagation operator of ASM for plane waves (see *Materials and methods*[Sec sec5.3] §5.3 for details). The phase shift and absorbance effects induced by target objects are applied by multiplying *U* by the exponential terms in equation (4). Using the ASM operator, the tensors for *o*_ϕ_ and *o*_*A*_ are linked across the depth range from *z*_1_ and *z*_*N*_. However, due to the memory limit of GPU, it is not feasible to keep all depth-wise tensors in the GPU memory and propagate *U* from *z*_N_ to the detector plane (*z*_0_ = 0). Instead, all object values between *z*_1_ and *z*_0_ are assumed to be 0 to perform free-space propagation as follows,
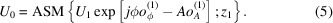
The MSE loss between the simulated intensity map |*U*_0_|^2^ and the input X-ray hologram *H* is then minimized to update model parameters of MorpHoloNet-X. Finally, phase and absorbance maps are obtained by element-wise multiplying the optimized ϕ and *A* coefficients by the corresponding object value arrays near the object plane, respectively.

### 2.2. Generation of shot-noise-limited synthetic holograms

Three absorbing and phase-shifting objects, denoted by the Greek letters α, β, and γ, are employed as reference data. The phase shift values of α, β, and γ are set to −0.3, −0.2, and −0.1 rad, respectively. The absorbance values of α, β, and γ are set to 0.03, 0.02, and 0.01, respectively. At the object plane, the phase shift and absorbance are applied to a 128 × 128 grid with a pixel size of 10 nm. Using the ASM operator, the complex wavefield is propagated by 100 µm to generate a noise-free hologram. The wavelength is set to 0.1327 nm for hologram simulation.

**Figure 2:**
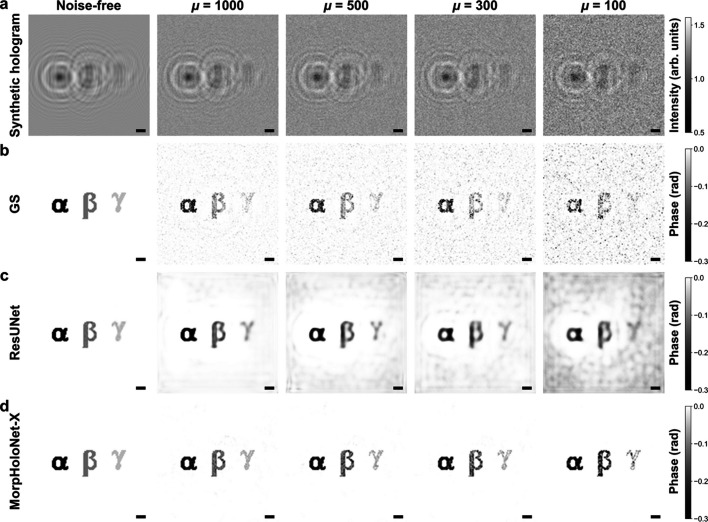
Comparison of the phase retrieval performance. (*a*) Noise-free synthetic hologram and its shot-noise-limited holograms. Phase maps reconstructed by (*b*) the Gerchberg–Saxton (GS) algorithm, (*c*) residual U-net (ResUNet), and (*d*) MorpHoloNet-X. Scale bars are 100 nm.

Shot-noise-limited (SNL) synthetic holograms are generated by applying the probability mass function (PMF) of the Poisson distribution to the noise-free hologram. At pixel (*p*, *q*) on the noise-free hologram *H*, the expected photon count can be expressed as follows,
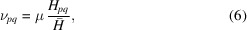
where 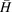 is the spatial average of *H* and μ denotes the mean photon count per pixel. For each pixel, the photon count*C*_*pq*_ is sampled by the PMF of the Poisson distribution as follows,
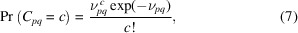
where *c* is a non-negative integer. Finally, the photon count at each pixel is rescaled to acquire an SNL hologram *H*′ as follows,
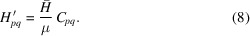
Fig. 2 ▸(*a*) shows the noise-free hologram and its SNL holograms simulated at μ = 1000, 500, 300, and 100.

### 2.3. Phase and absorbance retrieval from shot-noise-limited synthetic holograms

The phase and absorbance maps of synthetic holograms are reconstructed using the GS algorithm, ResUNet, and MorpHoloNet-X. In the phase maps reconstructed by the GS algorithm [Fig. 2 ▸(*b*)], the magnitude of the phase shift tends to be underestimated compared with the reference data. In addition, shot noise largely degrades phase retrieval performance for weak phase-shifting object γ. Although ResUNet exhibits better reconstruction performance than the GS algorithm, high-frequency edge structures are not fully recovered [Fig. 2 ▸(*c*)]. Furthermore, it tends to overfit the background as the number of epochs increases. By contrast, MorpHoloNet-X exhibits better edge preservation and robustness to background noise, compared with ResUNet [Fig. 2 ▸(*d*)].

**Figure 3:**
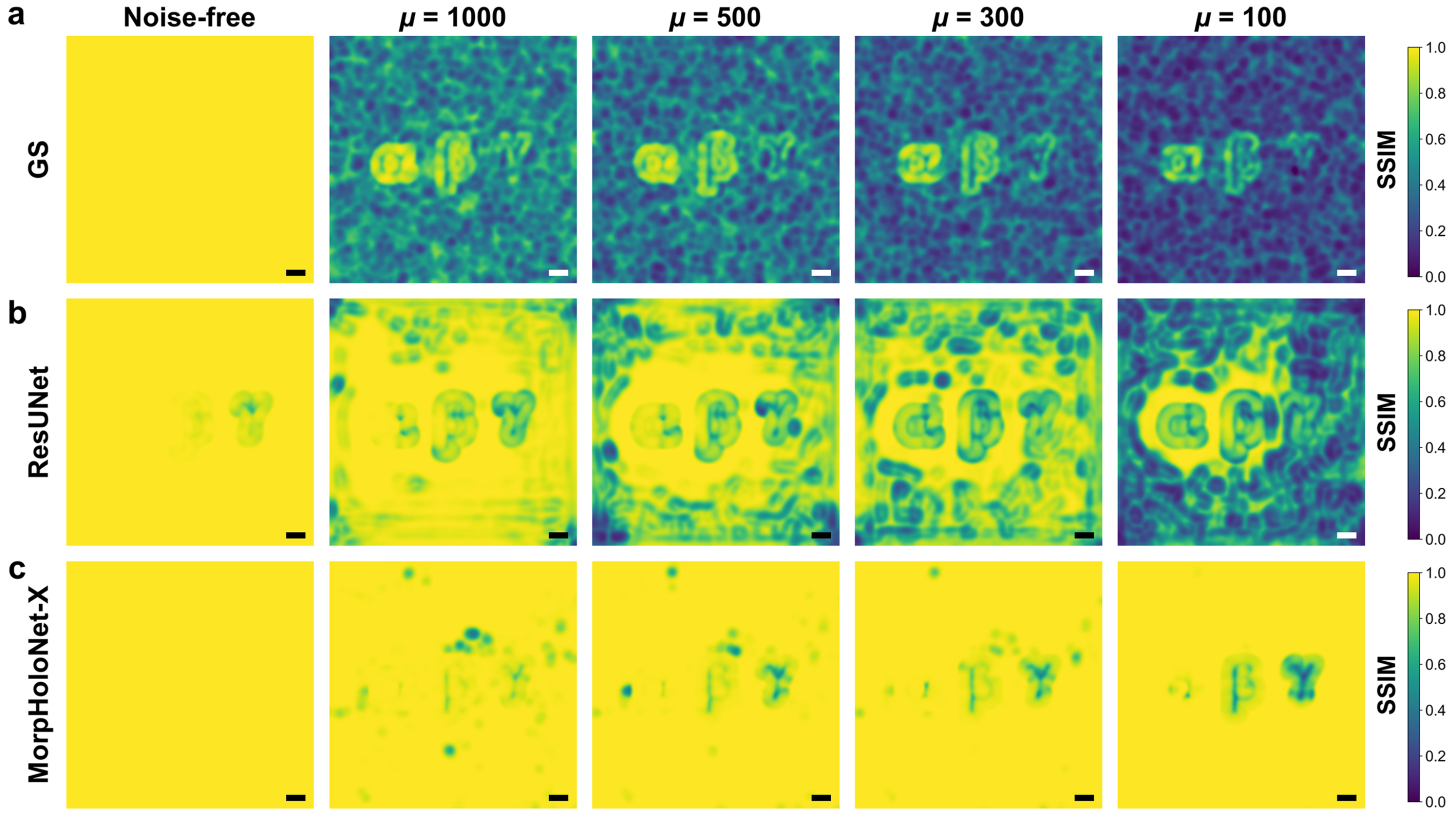
Structural similarity index measure (SSIM) maps for phase maps reconstructed from synthetic holograms using (*a*) the Gerchberg–Saxton (GS) algorithm, (*b*) residual U-Net (ResUNet), and (*c*) MorpHoloNet-X. Scale bars are 100 nm.

Fig. 3 ▸ shows structural similarity index measure (SSIM) maps computed by the reconstructed phase maps and the reference data (Wang *et al.*, 2004 ▸). Fig. S3 of the supporting information depicts SSIM profiles extracted along the horizontal centerline of the SSIM maps shown in Fig. 3 ▸. For μ ≥ 300, MorpHoloNet-X achieves higher SSIM values than the other phase retrieval methods. For μ = 100, the weak phase object γ exhibits a large reconstruction error. These results imply that, while MorpHoloNet-X is comparatively robust to shot noise and can recover accurate morphological features, its performance degrades when the mean photon count is below a certain threshold.

**Figure 4:**
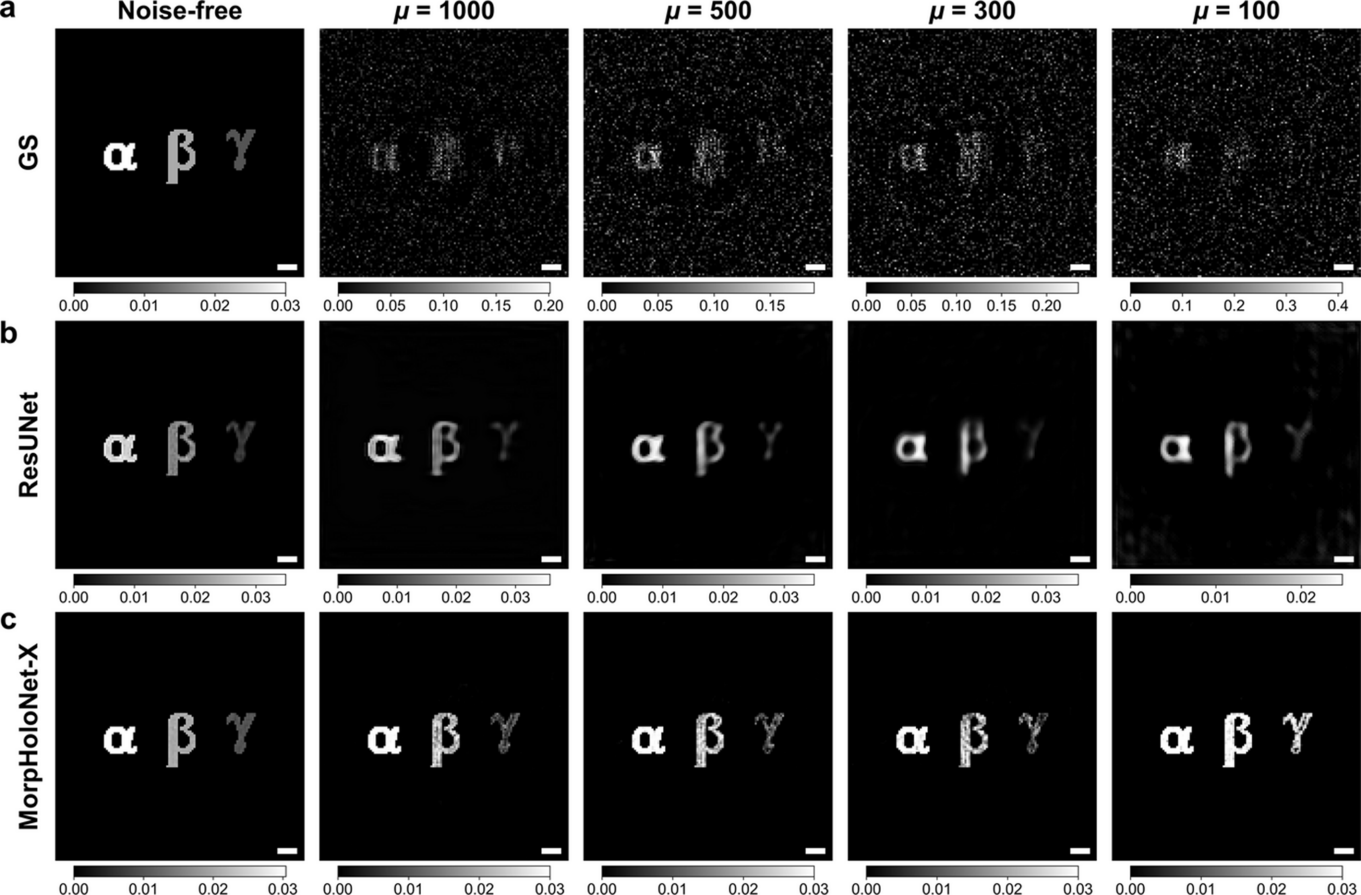
Comparison of absorbance retrieval performance. Absorbance maps reconstructed by (*a*) the Gerchberg–Saxton (GS) algorithm, (*b*) residual U-net (ResUNet), and (*c*) MorpHoloNet-X. Scale bars are 100 nm.

As shown in Fig. 4 ▸(*a*), the absorbance maps reconstructed by the GS algorithm from SNL holograms are inconsistent with the reference data. Accurate absorbance recovery depends on measuring variations in the amplitude of the propagating light. However, local amplitude fluctuations induced by shot noise hinder the convergence of the GS algorithm, resulting in relatively large reconstruction errors. Compared with the GS algorithm, ResUNet is more robust to shot noise and can recover the approximate shape of the absorbing objects [Fig. 4 ▸(*b*)]. The absorbance values obtained by ResUNet are generally underestimated relative to the reference data. In addition, the weakly absorbing object γ is recovered with low contrast. On the other hand, MorpHolo­Net-X provides absorbance values closer to the reference data than the other methods for μ ≥ 300 [Fig. 4 ▸(*c*)]. However, it still fails to accurately estimate the absorbance of the objects β and γ for μ = 100.

**Figure 5:**
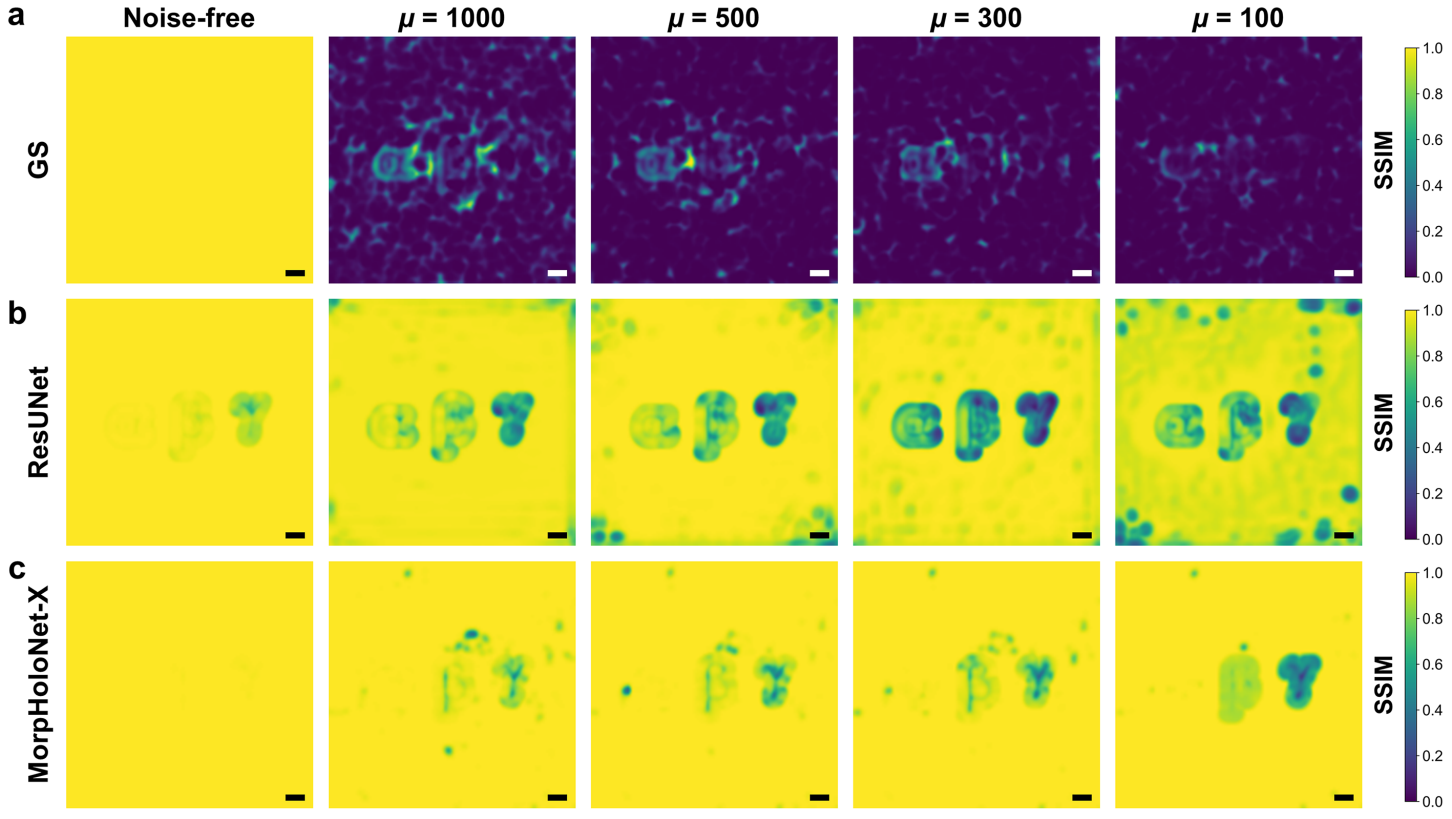
Structural similarity index measure (SSIM) maps for absorbance maps reconstructed from synthetic holograms using (*a*) the Gerchberg–Saxton (GS) algorithm, (*b*) residual U-Net (ResUNet), and (*c*) MorpHoloNet-X. Scale bars are 100 nm.

Fig. 5 ▸ shows SSIM maps comparing the reconstructed absorbance maps with the reference data. Fig. S4 of the supporting information presents SSIM profiles along the horizontal centerline of the SSIM maps shown in Fig. 5 ▸. The GS algorithm is unable to reconstruct any morphological information for the three absorbing objects from their SNL holograms. For ResUNet, the SSIM values for absorbance are somewhat lower than those for phase. Consistent with the SSIM values for phase, MorpHoloNet-X exhibits adequate morphological reconstruction for μ ≥ 300, whereas its performance deteriorates at μ = 100.

**Figure 6:**
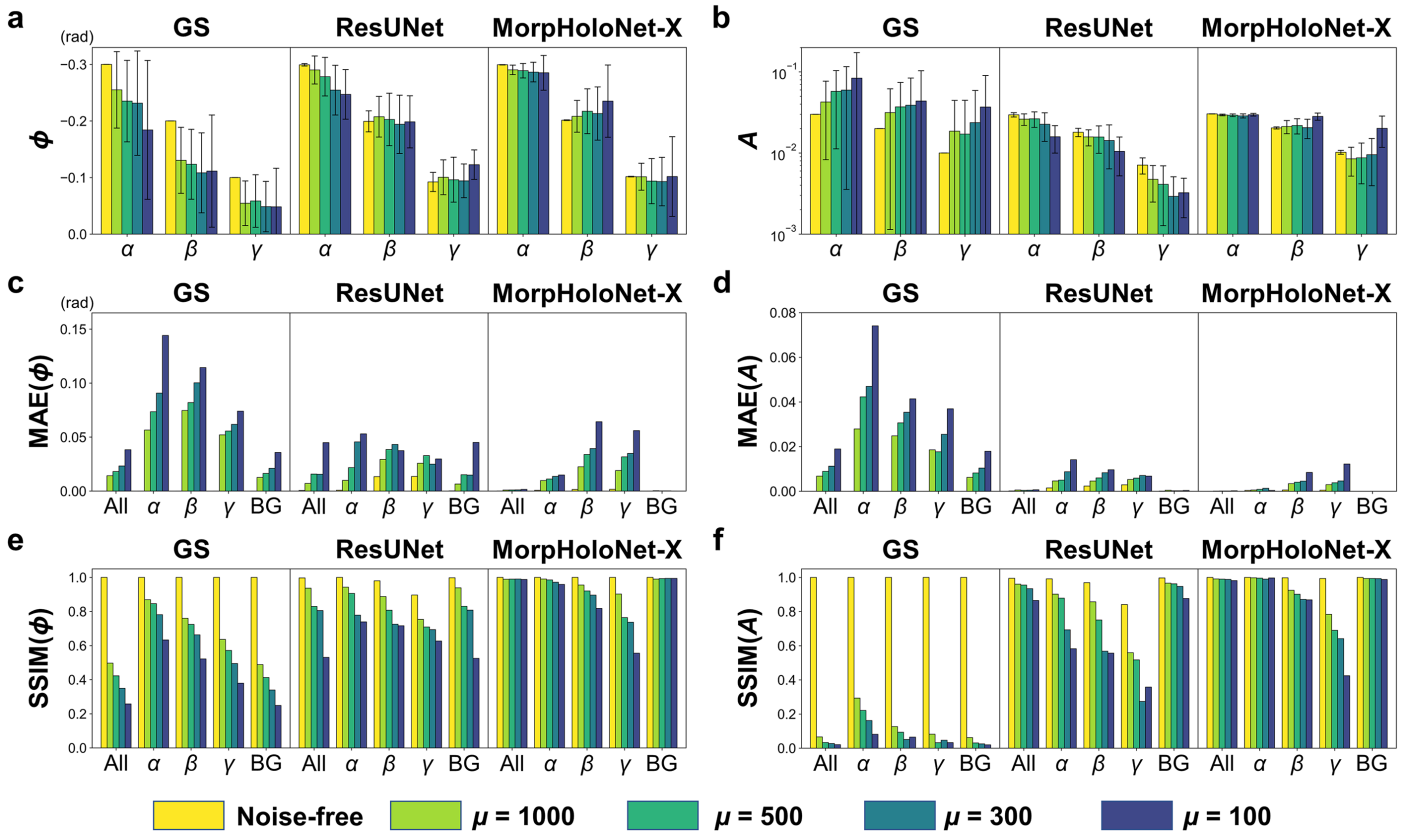
Comparisons of phase and absorbance retrieval performance of the Gerchberg–Saxton (GS) algorithm, residual U-Net (ResUNet), and MorpHoloNet-X from synthetic holograms. (*a*) Mean phase shift and (*b*) mean absorbance values of absorbing and phase-shifting objects α, β, and γ. Mean absolute errors (MAEs) of (*c*) phase shift and (*d*) absorbance, evaluated between the reconstructed results and the reference data. Structural similarity index measure (SSIM) values of (*e*) phase shift and (*f*) absorbance, evaluated between the reconstructed results and the reference data.

Fig. 6 ▸ shows quantitative evaluations of phase and absorbance retrieval performance for the GS algorithm, ResUNet, and MorpHoloNet-X. The mean, mean absolute error (MAE), and SSIM of phase and absorbance are analyzed in five separated regions, including the entire hologram, the target objects (α, β, and γ), and the background. Overall, the GS algorithm exhibits perfect reconstruction performance for the noise-free hologram. The magnitude of phase shift values reconstructed by the GS algorithm is increasingly underestimated as μ decreases. In particular, the reconstructed absorbance exhibits abnormally large values compared with the reference data. In addition, for both phase and absorbance, the corresponding MAE values increase and the SSIM values decrease as μ decreases. This result indicates that the GS algorithm is not suitable for analyzing SNL holograms.

For the case of ResUNet, the phase information of β and γ is well reconstructed, whereas the phase of α tends to be underestimated due to shot noise. The reconstructed absorbance is increasingly underestimated for all target objects as μ decreases. Compared with the GS algorithm, ResUNet outperforms in phase and absorbance retrieval for SNL holograms. For phase values of the GS algorithm, the average percentage errors are 31.8, 33.8, 40.0, and 44.9% for μ = 1000, 500, 300, and 100, respectively. For phase values of ResUNet, the average percentage errors are 2.5, 4.1, 8.0, and 13.7% for μ = 1000, 500, 300, and 100, respectively. For absorbance values of the GS algorithm, the average percentage errors are 61.7, 82.7, 110.1, 189.1% for μ = 1000, 500, 300, and 100, respectively. For absorbance values of ResUNet, the average percentage errors are 28.9, 30.7, 41.4, 54.1% for μ = 1000, 500, 300, and 100, respectively.

MorpHoloNet-X generally outperforms the other methods in both phase and absorbance retrieval for SNL holograms. For phase values of MorpHoloNet-X, the average percentage errors are 2.9, 6.2, 6.1, and 8.1% for μ = 1000, 500, 300, and 100, respectively. For absorbance values of MorpHoloNet-X, the average percentage errors are 7.7, 8.2, 4.0, and 47.9% for μ = 1000, 500, 300, and 100, respectively. Except for the case of γ at μ = 100, MorpHoloNet-X achieves higher SSIM values than ResUNet. This implies that MorpHoloNet-X better recovers morphological features of the target objects. On the other hand, for μ = 100, the percentage errors in absorbance for β and γ are 41.0 and 101.3%, respectively. The SSIM of MorpHoloNet-X for γ at μ = 100 is 11.3% lower than that of ResUNet. Since the performance of AI-based phase and absorbance reconstruction degrades at μ = 100, it is practically challenging to solve an inverse problem for accurate phase and absorbance retrieval from holograms contaminated by severe shot noise.

### 2.4. Phase and absorbance retrieval from a hard X-ray hologram

**Figure 7:**
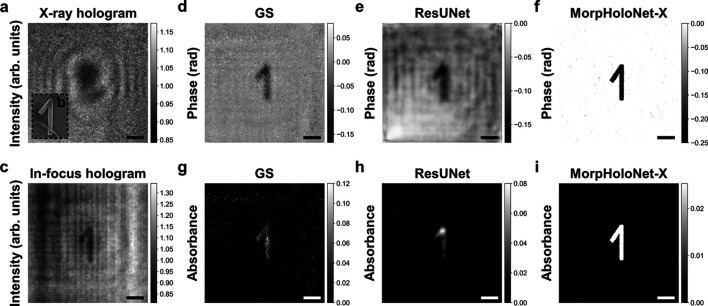
Phase and absorbance retrieval from a hard X-ray hologram. (*a*) Intensity map of the X-ray hologram and (*b*) scanning electron microscope image of the target sample. (*c*) In-focus hologram reconstructed using the angular spectrum method. (*d*–*f*) Phase shift maps reconstructed by the Gerchberg–Saxton (GS) algorithm, residual U-Net (ResUNet), and MorpHoloNet-X. (*g*–*i*) Absorbance maps reconstructed by the GS algorithm, ResUNet, and MorpHoloNet-X. Scale bars are 1 µm.

Fig. 7 ▸(*a*) shows a hard X-ray hologram of the digit ‘1’ on a resolution target, acquired at the 7C XNI beamline of PLS-II (see *Materials and methods*[Sec sec5.1] §5.1 for details). The digit ‘1’ is a gold pattern with a thickness of 170 nm deposited on a silicon nitride membrane. The ground-truth phase shift and absorbance induced by the target sample are −0.278 rad and 0.023, respectively. The scanning electron microscope (SEM) image [Fig. 7 ▸(*b*)] and the in-focus hologram reconstructed by the ASM [Fig. 7 ▸(*c*)] exhibit similar morphology. The object mask label for training MorpHoloNet-X is obtained by edge-based segmentation of the in-focus hologram [see *Method* §S1 and Fig. S1(*b*) of the supporting information]. Phase and absorbance maps of the target sample are recovered from the hard X-ray hologram using the GS algorithm, ResUNet, and MorpHoloNet-X [Figs. 7 ▸(*d*)–7(*i*)].

The phase shift values reconstructed by the GS algorithm, ResUNet, and MorpHoloNet-X are −0.066 ± 0.033, −0.129 ± 0.018, and −0.240 ± 0.027 rad, respectively. The corresponding percentage errors of phase retrieval are 76.2, 53.7, and 13.5%, respectively. The absorbance values reconstructed by the GS algorithm, ResUNet, and MorpHoloNet-X are 0.034 ± 0.024, 0.021 ± 0.007, and 0.025 ± 0.002 rad, respectively. The corresponding percentage errors of absorbance retrieval are 50.7, 7.2, and 9.7%, respectively. Although the mean absorbance reconstructed by ResUNet is the most accurate, it fails to properly recover high-frequency morphological features at the edges. By contrast, since such edge structures are inferable from the in-focus hologram, this prior knowledge can be adopted to pre-train MorpHoloNet-X. These results indicate that MorpHoloNet-X can serve as an alternative approach to solving an inverse problem of single-shot phase and absorbance retrieval from hard X-ray holograms under weak illumination.

## 3. Discussion

The proposed MorpHoloNet-X benefits from incorporating the physics-based prior knowledge into the training process of neural networks. For example, undesirable artifacts induced by shot noise during hologram reconstruction can be suppressed by adopting the proposed pre-training process. This approach reduces unnecessary reconstruction errors in the background region at the object plane [Figs. 6 ▸(*c*) and 6(*d*)]. Since the intensity map obtained by the ASM contains 2D shapes of target objects along with twin image artifacts, this morphological information can be used to train MorpHolo­Net-X by adopting object mask labels. For small holograms, vertical and horizontal fringe patterns generated near the image boundaries are reconstructed together at the object plane during wave propagation [Figs. 7 ▸(*c*)–7(*e*)]. These line artifacts can be addressed by imposing boundary conditions to force the object values at the image boundaries to be zero. Since shot noise diminishes high-frequency components of fine holographic interference signals and induces attenuation fluctuations, it is difficult to recover intact physical information from SNL holograms. By using prior knowledge of specimens to initialize voxel-wise phase and absorbance, MorpHoloNet-X can converge to a local optimum near the ground truth solution.

There are several limitations in practical XHM application of MorpHoloNet-X. For object mask label generation, it is helpful to perform segmentation with the aid of auxiliary SEM images of specimens. To initialize voxel-wise phase and absorbance, the chemical components and optical properties of the specimens should be known in advance of X-ray experiments. Incorporating such prior knowledge into MorpHoloNet-X seems cumbersome. However, under SNL conditions imposed by unavoidable hardware constraints, it is necessary to employ prior knowledge obtained through sample characterization to reduce measurement errors in phase and absorbance retrieval. Since an excessive number of epochs leads to overfitting to shot noise, it is required to apply early stopping at the point where shot-noise patterns begin to appear in the reconstructed object values.

Although the incident illumination is cone-beam, the ASM for plane waves is employed for wave propagation. This assumption is intended to connect tensor arrays at adjacent depths using the ASM operator to simplify the optimization of MorpHoloNet-X. Nevertheless, this connection may contribute to reconstruction errors in the experimental results reported in this study. Since the lateral resolution of the present XHM setup is limited to 213 nm at the 7C XNI beamline (see *Materials and methods*[Sec sec5.1] §5.1 for details), it is impossible to perform additional validation on finer structures of specimens. To further improve the lateral resolution, the focal spot should be reduced by using a smaller waveguide channel. However, the photon flux density at the waveguide exit becomes extremely low, leading to a severely reduced SNR. For further improvements, the optical setup needs to be upgraded with a better focusing optical system for increasing photon flux density.

With a higher incident photon flux density, the proposed MorpHoloNet-X could be broadly utilized in various nanotomographic studies. Due to the low photon flux density in the present setup, holograms are acquired with an exposure time of 10 s. If the exposure time can be shortened with sufficient photon flux, MorpHoloNet-X would enable the analysis of rapid morphological and compositional changes through single-shot phase and absorbance retrieval. In our previous work (Kim *et al.*, 2025 ▸), the original MorpHoloNet was used to reconstruct the 3D morphology of biological cells from their single-shot hologram. To apply this concept to XHM, clear holographic interference signals are essential. However, it is challenging to experimentally validate 3D morphology reconstruction performance from SNL holograms obtained in the present setup. If 3D morphology reconstruction of MorpHoloNet-X can be demonstrated using clear hard X-ray holograms, the acquisition time for morphological analysis of specimens could be further reduced.

## 4. Conclusions

In summary, an AI-based XHM technique is developed for single-shot phase and absorbance retrieval from SNL hard X-ray holograms using a physics-driven neural network. The performance of phase and absorbance reconstruction using MorpHoloNet-X is evaluated using synthetic and experimental holograms and compared with that of the GS algorithm and ResUNet. The proposed technique can recover physical information suppressed by shot noise by solving an inverse problem with physics-based prior knowledge. MorpHoloNet-X would be utilized to reconstruct phase and absorbance information from SNL hard X-ray holograms obtained under fast acquisition or weak illumination.

## 5. Materials and methods

### 5.1. Optical setup of X-ray holographic microscopy

**Figure 8:**
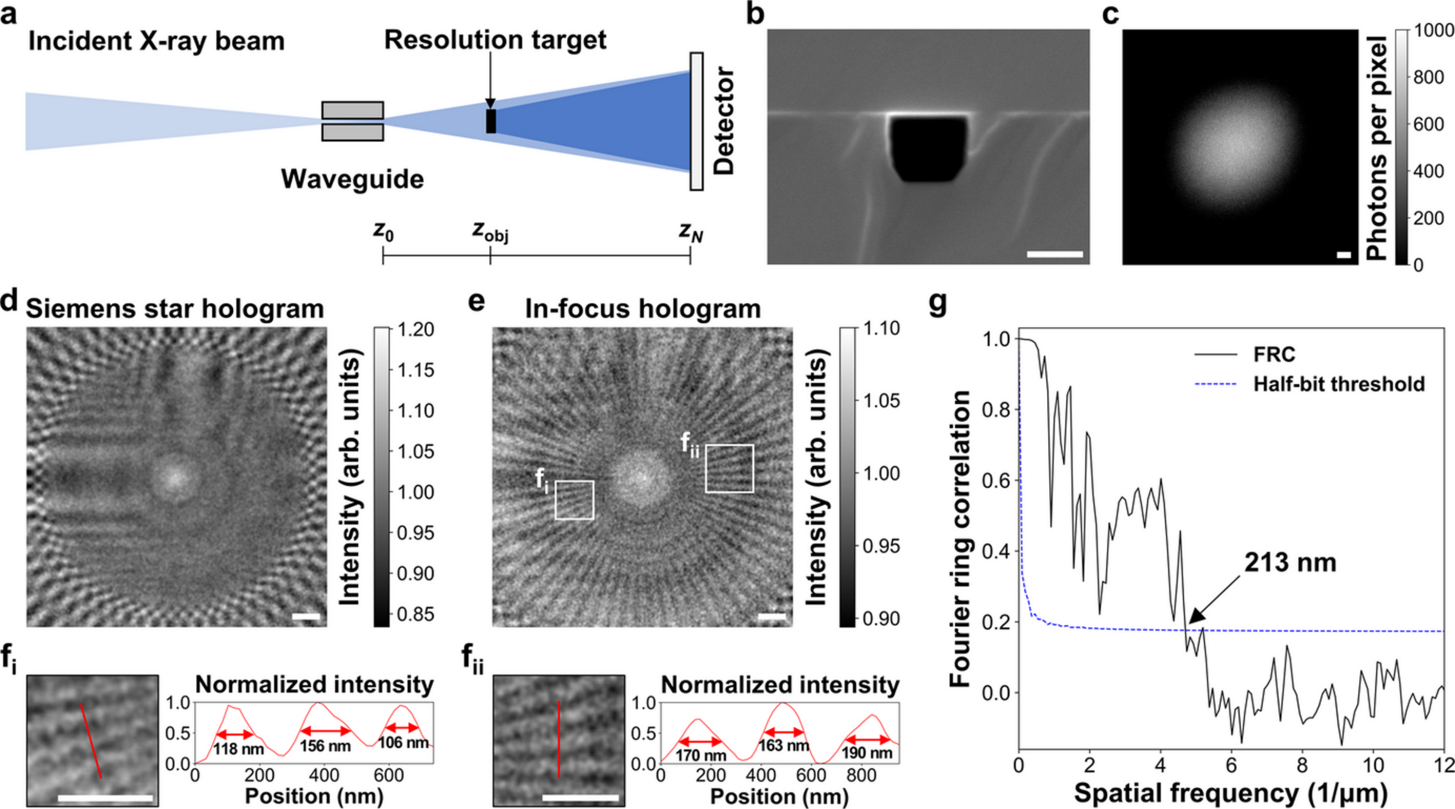
X-ray holographic microscopy (XHM) setup. (*a*) Schematic of the XHM optical setup with (*b*) a waveguide channel. (*c*) Background image and (*d*) Siemens star hologram recorded with an exposure time of 10 s using the XHM setup. (*e*) Reconstructed in-focus hologram. (*f*_i,ii_) Resolved half-pitch features. (*g*) Fourier ring correlation (FRC) analysis. Scale bars: (*b*) 100 nm, (*c*–*f*) 1 µm.

Hard X-ray holograms were acquired at the 7C XNI beamline at PLS-II [Fig. 8 ▸(*a*)]. Since the X-ray beam energy is set to 9.344 keV at the 7C XNI beamline, the corresponding wavelength is 0.1327 nm. The optical setup of XHM consisted of an undulator source, a liquid-nitro­gen-cooled silicon (111) double-crystal monochromator (Vactron, Korea), a beryllium parabolic CRL (RXOPTICS GmbH, Germany), a custom-made waveguide channel of 140 nm × 120 nm [Fig. 8 ▸(*b*)], a Ce:GAGG scintillator (thickness = 20 µm), a 20× objective lens (LD Plan-Neofluar 20×/0.4 Corr M27, Zeiss, USA), and a scientific complementary metal oxide semiconductor (sCMOS) camera (01-KINETIX-M-C, Teledyne, USA; 3200 × 3200 pixels, pixel size of 6.5 µm × 6.5 µm).

The 2D waveguide was mounted on a six-axis motorized translation-and-rotation stage to precisely align its nanoscale waveguide channel with the incident X-ray beam at the focal spot. The strong light signal from a large waveguide channel was identified first. Subsequently, the signals from adjacent smaller channels were identified step by step. A resolution target (Applied Nanotools, Canada) was attached on a three-axis motorized translation stage to acquire its holograms. The waveguide–detector distance (*z*_*N*_–*z*_0_) was 15 cm, and the waveguide–object distance (*z*_obj_–*z*_0_) was 8 mm. The magnified pixel size on the object plane (*z* = *z*_obj_) was measured to be 17.15 nm. The exposure time for the sCMOS camera was set to 10 s.

Fig. 8 ▸(*c*) shows the background image recorded by the sCMOS camera. The recorded photons per pixel in the background image were consistent with the μ range set for the SNL synthetic holograms. A hard X-ray hologram of a Siemens star pattern on the resolution target was acquired to evaluate the lateral resolution of the XHM setup [Fig. 8 ▸(*d*)]. The ASM was employed to reconstruct its in-focus hologram at the object plane [Fig. 8 ▸(*e*)]. Half-pitch features of approximately 130 nm were resolved in the reconstructed in-focus hologram [Fig. 8 ▸(*f*_i_), 8(*f*_ii_)]. Fourier ring correlation (Nieuwenhuizen *et al.*, 2013 ▸) was utilized to calculate the lateral resolution from two independent in-focus holograms of the Siemens star pattern, yielding a resolution of 213 nm [Fig. 8 ▸(*g*)].

### 5.2. Waveguide fabrication

A 2D waveguide was fabricated at National NanoFab Center and Korea Advanced Nano Fab Center in Korea by following the methodology described in Salditt’s work (Neubauer *et al.*, 2014 ▸). High-aspect-ratio X-ray waveguide channels were patterned in positive e-beam resist (thickness 200 nm) on a silicon wafer (thickness 725 µm) using an e-beam lithography system (JBX-9300FS, Jeol, Japan). The widths of the X-ray waveguide channels were 20, 10, 5, 1, 0.2, and 0.1 µm with a fixed length of 40 mm. The e-beam patterns were dry etched to a depth of 100 nm using a poly etcher (TCP-9400DFM, Lam Research, USA). The etched wafer was then bonded with a bare silicon wafer using a fusion bonder (EVG520HE, EV Group, Austria). The bonded wafer was cut to a length of 2 mm using a wafer dicing machine (DFD6340, DISCO, Japan). To prevent blockage of the waveguide channels by a dicing blade, scribe lines with a depth of 600 µm were formed on both faces of the bonded wafer before cleaving. Finally, a nanoscale waveguide channel with cross-sectional dimensions of 140 nm × 120 nm was fabricated [Fig. 8 ▸(*b*)].

### 5.3. Wave propagation using the angular spectrum method

The propagation of a complex wavefield (*U*) was simulated along the *z*-axis in steps of Δ*z* by using the ASM as follows,
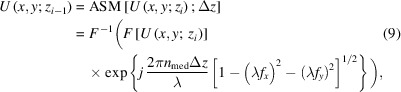
where *x* and *y* denote the spatial coordinates on a rectangular grid of *L* × *M* pixels (Goodman, 2005 ▸; Choi *et al.*, 2012 ▸). *f*_*x*_ and *f*_*y*_ represent the spatial frequencies corresponding to the *x* and *y* coordinates, respectively. *n*_med_ is the refractive index in a medium. *F* and *F*^−1^ indicate the fast Fourier transform and inverse fast Fourier transform, respectively.

### 5.4. Network architectures of MorpHoloNet-X

The model architecture of MorpHoloNet-X was slightly modified from the original MorpHoloNet in our previous study (Kim *et al.*, 2025 ▸). MorpHoloNet-X is a fully connected network NN(*x*, *y*, *z*; *w*, *b*) composed of one input layer with normalization, one Fourier feature projection layer for positional encoding (Tancik *et al.*, 2020 ▸), three dense layers, and one output layer [Fig. 1 ▸(*b*)]. *w* and *b* denote the weights and biases within MorpHoloNet-X. The in-plane coordinates (*x*, *y*) were normalized by *L*Δ*x* and *M*Δ*y*, respectively. The pixel size of Δ*x* and Δ*y* was set to 10 nm for the synthetic holograms and 34.3 nm for the experimental X-ray hologram. The axial coordinate (*z*) was normalized as 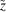 = 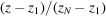 near the approximate depth *z*′. The depth interval (Δ*z*) was 20 nm for the synthetic holograms and 80 nm for the experimental X-ray hologram. The normalized 3D coordinates 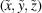 ranged from 0 and 1. The Fourier feature projection layer and each of the three dense layers had an output size of 128. The Gaussian scale for positional encoding was set to 15 for the synthetic holograms and 25 for the experimental hologram. The dense layers used the swish activation functions (Ramachandran *et al.*, 2017 ▸). The output layer employed the sigmoid activation functions.

For pre-training, the MSE loss between the object value outputs (*o*_ϕ_ and *o*_*A*_) and the corresponding object mask labels (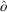) was minimized using the Adam optimizer [Fig. 1 ▸(*c*)] (Kingma & Ba, 2014 ▸). The training loss from prior knowledge (*L*_Prior_) was defined as follows,
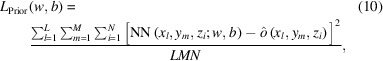
where *x*_*l*_ and *y*_*m*_ denote the discretized spatial coordinates on the 2D complex wavefield at each depth *z*_*i*_. After pre-training, the physics-based forward model for wave propagation of the ASM and boundary conditions were utilized to train MorpHoloNet-X. The training losses for the boundary conditions on the six faces of the 3D volume were defined as follows,
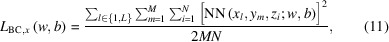

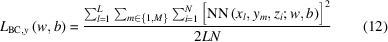

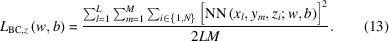
The total training loss for the boundary conditions (*L*_BC_) was evaluated by summing *L*_BC,*x*_, *L*_BC,*y*_, and *L*_BC,*z*_ [Fig. 1 ▸(*d*)].

The training loss for the wave propagation using the ASM is defined as follows,
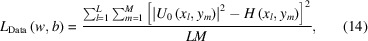
where |*U*_0_|^2^ and *H* denote the simulated and experimental intensity maps at the object plane (*z*_0_ = 0), respectively. The image size of *H* was 128 × 128 pixels for the synthetic holograms and 200 × 200 pixels for the experimental X-ray hologram. Finally, the summation of *L*_Data_ and *L*_BC_ was minimized using the Adam optimizer for phase and absorbance retrieval. The ϕ and *A* coefficients were kept fixed for the synthetic holograms to enable a straightforward comparison with ResUNet. In contrast, they were treated as trainable parameters for the experimental hologram because their true values were not known in advance. The pre-training process was performed for 300 epochs with a learning rate of 10^−3^. For the synthetic holograms, the number of epochs and learning rate for phase and absorbance retrieval were set to 3000 and 10^−4^, respectively. For the experimental X-ray hologram, the number of epochs and learning rate for phase and absorbance retrieval were set to 2000 and 10^−5^, respectively. The hyperparameters can be adjusted through fine-tuning.

### 5.5. Network architectures of ResUNet

The ResUNet architecture consisted of a residual encoder–decoder network with three downsampling stages, symmetric upsampling stages, two output layers, and skip connections (Zhang *et al.*, 2018 ▸). Swish activation functions were applied to both encoder and decoder. At the first output layer, a phase map was predicted with a 1 × 1 convolution with a sigmoid activation function. The predicted phase map was scaled by 0.3 so that the final phase values ranged from 0 to 0.3. This scaling step was adopted to reflect the maximum magnitude of phase shift values for the synthetic holograms (0.3 rad) and the experimental X-ray hologram (0.278 rad). The second output layer was fed by feature maps of the predicted phase map through an additional residual block layer. An absorbance map was predicted with a 1 × 1 convolution with a leaky rectified linear unit activation function (Maas *et al.*, 2013 ▸). The predicted absorbance map was scaled by 0.03 for the synthetic holograms and 0.1 for the experimental X-ray hologram. The scaled phase and absorbance maps at the object plane were forward-propagated to the detector plane using the ASM. The MSE loss between the simulated and input holograms was minimized to train ResUNet. The number of epochs was set to 3 × 10^4^ for the synthetic holograms and 3 × 10^5^ for the experimental X-ray hologram. The learning rate was set to 10^−6^.

### 5.6. Development environment

MorpHoloNet-X training was performed under Python 3.6.7, Anaconda3-4.5.11, PyCharm (JetBrains, Czech Republic), TensorFlow-gpu 2.4.1, NVIDIA CUDA toolkit 11.0, and cuDNN 8.2.1. A workstation was equipped with Nvidia GeForce RTX 3090 GPU, AMD Ryzen 5950X CPU, and 128 GB RAM. MATLAB R2021a *ImageJ* software programs were utilized to conduct digital image processing of captured holograms.

## Supplementary Material

Supporting information including Method S1 and Figures S1-S4. DOI: 10.1107/S1600577526003188/mo5317sup1.pdf

## Data Availability

Data underlying the results presented in this paper are available from the authors upon reasonable request. Source code of MorpHoloNet-X is available at https://github.com/Holomolu/MorpHoloNet-X.

## References

[bb1] Abbey, B., Nugent, K. A., Williams, G. J., Clark, J. N., Peele, A. G., Pfeifer, M. A., de Jonge, M. & McNulty, I. (2008). *Nat. Phys.* **4**, 394–398.

[bb2] Aoki, S. & Kikuta, S. (1974). *Jpn. J. Appl. Phys.* **13**, 1385–1392.

[bb3] Bartels, M., Krenkel, M., Haber, J., Wilke, R. N. & Salditt, T. (2015). *Phys. Rev. Lett.* **114**, 048103. 10.1103/PhysRevLett.114.048103 25679911

[bb4] Charrière, F., Colomb, T., Montfort, F., Cuche, E., Marquet, P. & Depeursinge, C. (2006). *Appl. Opt.* **45**, 7667–7673. 10.1364/ao.45.007667 17068602

[bb5] Charriére, F., Rappaz, B., Kühn, J., Colomb, T., Marquet, P. & Depeursinge, C. (2007). *Opt. Express* **15**, 8818–8831. 10.1364/oe.15.008818 19547218

[bb6] Cheung, M., Adaniya, H., Cassidy, C., Yamashita, M. & Shintake, T. (2020). *Ultramicroscopy* **209**, 112883. 10.1016/j.ultramic.2019.112883 31739191

[bb7] Choi, Y. S., Seo, K. W., Sohn, M. H. & Lee, S. J. (2012). *Opt. Lasers Eng.* **50**, 39–45.

[bb8] Croton, L. C., Morgan, K. S., Paganin, D. M., Kerr, L. T., Wallace, M. J., Crossley, K. J., Miller, S. L., Yagi, N., Uesugi, K., Hooper, S. B. & Kitchen, M. J. (2018). *Sci. Rep.* **8**, 11412. 10.1038/s41598-018-29841-5 PMC6065359 30061729

[bb9] Deng, M., Li, S., Goy, A., Kang, I. & Barbastathis, G. (2020). *Light Sci. Appl.* **9**, 36. 10.1038/s41377-020-0267-2 PMC7062747 32194950

[bb10] Faigel, G. & Tegze, M. (1999). *Rep. Prog. Phys.* **62**, 355–393.

[bb11] Gabor, D. (1948). *Nature* **161**, 777–778. 10.1038/161777a0 18860291

[bb12] Gerchberg, R. W. & Saxton, W. O. (1972). *Optik* **35**, 237–246.

[bb13] Glaeser, R. M. (2013). *Rev. Sci. Instrum.* **84**, 111101. 10.1063/1.4830355 PMC3855062 24289381

[bb14] Goodman, J. W. (2005). *Introduction to Fourier Optics.* Roberts and Company.

[bb15] Gureyev, T. E., Davis, T. J., Pogany, A., Mayo, S. C. & Wilkins, S. W. (2004). *Appl. Opt.* **43**, 2418–2430. 10.1364/ao.43.002418 15119611

[bb16] Heine, R., Gorniak, T., Nisius, T., Christophis, C., Pettitt, M., Staier, F., Wilhein, T., Rehbein, S., Grunze, M. & Rosenhahn, A. (2011). *Ultramicroscopy* **111**, 1131–1136. 10.1016/j.ultramic.2011.02.002 21740876

[bb17] Jacobsen, C., Howells, M., Kirz, J. & Rothman, S. (1990). *J. Opt. Soc. Am. A* **7**, 1847–1861.

[bb18] Jo, S., Kim, H., Kim, S., Nam, C., Lim, J. & Lim, J. (2025). *Small Methods* **9**, 2401087. 10.1002/smtd.202401087 39659129

[bb19] Kalbfleisch, S., Zhang, Y., Kahnt, M., Buakor, K., Langer, M., Dreier, T., Dierks, H., Stjärneblad, P., Larsson, E., Gordeyeva, K., Chayanun, L., Söderberg, D., Wallentin, J., Bech, M. & Villanueva-Perez, P. (2022). *J. Synchrotron Rad.* **29**, 224–229. 10.1107/S1600577521012200 PMC8733976 34985439

[bb20] Kang, I., Zhang, F. & Barbastathis, G. (2020). *Opt. Express* **28**, 21578–21600. 10.1364/OE.397430 32752433

[bb21] Kim, J., Kim, Y., Lee, H. S., Seo, E. & Lee, S. J. (2025). *Nat. Commun.* **16**, 4840. 10.1038/s41467-025-60200-x PMC12103610 40413181

[bb22] Kingma, D. P. & Ba, J. (2014). *arXiv*:1412.6980.

[bb23] Kitchen, M. J., Lewis, R., Yagi, N., Uesugi, K., Paganin, D., Hooper, S. B., Adams, G., Jureczek, S., Singh, J., Christensen, C., Hufton, A. P., Hall, C. J., Cheung, K. C. & PAvlov, K. M. (2005). *Br. J. Radiol.* **78**, 1018–1027. 10.1259/bjr/13024611 16249603

[bb24] Kuan, A. T., Phelps, J. S., Thomas, L. A., Nguyen, T. M., Han, J., Chen, C. L., Azevedo, A. W., Tuthill, J. C., Funke, J., Cloetens, P., Pacureanu, A. & Lee, W. A. (2020). *Nat. Neurosci.* **23**, 1637–1643. 10.1038/s41593-020-0704-9 PMC8354006 32929244

[bb25] Latychevskaia, T. (2019). *J. Opt. Soc. Am. A* **36**, D31–D40. 10.1364/JOSAA.36.000D31 31873366

[bb26] Latychevskaia, T. & Fink, H. W. (2007). *Phys. Rev. Lett.* **98**, 233901. 10.1103/PhysRevLett.98.233901 17677906

[bb27] Latychevskaia, T. & Fink, H. W. (2015). *Appl. Opt.* **54**, 2424–2434. 10.1364/AO.54.002424 25968531

[bb28] Lesaffre, M., Verrier, N. & Gross, M. (2012). *Appl. Opt.* **52**, A81–A91. 10.1364/AO.52.000A81 23292425

[bb29] Lim, J., Kim, H. & Park, S. Y. (2014). *J. Synchrotron Rad.* **21**, 827–831. 10.1107/S1600577514008224 24971982

[bb30] Lim, J., Park, S. Y., Huang, J. Y., Han, S. M. & Kim, H. T. (2013). *Rev. Sci. Instrum.* **84**, 013707. 10.1063/1.4789362 23387659

[bb31] Lim, J., Shin, H. J. & Hong, C. K. (2011). *Jpn. J. Appl. Phys.* **50**, 072504.

[bb32] Liu, R., Sun, Y., Zhu, J., Tian, L. & Kamilov, U. S. (2022). *Nat. Mach. Intell.* **4**, 781–791.

[bb33] Maas, A. L., Hannun, A. Y. & Ng, A. Y. (2013). *Proceedings of the 30th International Conference on Machine Learning*, Atlanta, Georgia, USA,

[bb34] McNulty, I., Kirz, J., Jacobsen, C., Anderson, E. H., Howells, M. R. & Kern, D. P. (1992). *Science* **256**, 1009–1012. 10.1126/science.256.5059.1009 17795006

[bb35] Mom, K., Langer, M. & Sixou, B. (2023). *Opt. Lett.* **48**, 1136–1139. 10.1364/OL.484862 36857232

[bb36] Mom, K., Sixou, B. & Langer, M. (2022). *Appl. Opt.* **61**, 2497–2505. 10.1364/AO.443330 35471314

[bb37] Neubauer, H., Hoffmann, S., Kanbach, M., Haber, J., Kalbfleisch, S., Krüger, S. & Salditt, T. (2014). *J. Appl. Phys.* **115**, 214305.

[bb38] Nieuwenhuizen, R. P., Lidke, K. A., Bates, M., Puig, D. L., Grünwald, D., Stallinga, S. & Rieger, B. (2013). *Nat. Methods* **10**, 557–562. 10.1038/nmeth.2448 PMC4149789 23624665

[bb39] Ramachandran, P., Zoph, B. & Le, Q. V. (2017). *arXiv*:1710.05941.

[bb40] Rogalski, M., Arcab, P., Wdowiak, E., Picazo-Bueno, J. Á., Micó, V., Jozwik, M. & Trusiak, M. (2025). *ACS Photon.* **12**, 1771–1782. 10.1021/acsphotonics.4c01863 PMC12007103 40255508

[bb41] Soltau, J., Vassholz, M., Osterhoff, M. & Salditt, T. (2021). *Optica* **8**, 818–823.

[bb42] Tancik, M., Srinivasan, P., Mildenhall, B., Fridovich-Keil, S., Raghavan, N., Singhal, U., Ramamoorthi, R., Barron, J. & Ng, R. (2020). *Proceedings of the 34th International Conference on Neural Information Processing Systems (NIPS’20)*, pp. 7537–7547.

[bb43] Teague, M. R. (1983). *J. Opt. Soc. Am.* **73**, 1434–1441.

[bb44] Wang, F., Bian, Y., Wang, H., Lyu, M., Pedrini, G., Osten, W., Barbastathis, G. & Situ, G. (2020). *Light Sci. Appl.* **9**, 77. 10.1038/s41377-020-0302-3 PMC7200792 32411362

[bb45] Wang, K., Song, L., Wang, C., Ren, Z., Zhao, G., Dou, J., Di, J., Barbastathis, G., Zhou, R. & Zhao, J. (2024). *Light Sci. Appl.* **13**, 4. 10.1038/s41377-023-01340-x PMC10758000 38161203

[bb46] Wang, Z., Bovik, A. C., Sheikh, H. R. & Simoncelli, E. P. (2004). *IEEE Trans. Image Process.* **13**, 600–612. 10.1109/tip.2003.819861 15376593

[bb47] Xie, Y., Takikawa, T., Saito, S., Litany, O., Yan, S., Khan, N., Tombari, F., Tompkin, J., sitzmann, V. & Sridhar, S. (2022). *Comput. Graph. Forum* **41**, 641–676.

[bb48] Yang, X., Hailu, D., Kulvait, V., Jentschke, T., Flenner, S., Greving, I., Campbell, S. I., Hagemann, J., Schroer, C. G., Wong, T. M. & Moosmann, J. (2025). *Opt. Express* **33**, 35832–35851. 10.1364/OE.569216 40984363

[bb49] Zhang, F., Liu, X., Guo, C., Lin, S., Jiang, J. & Ji, X. (2021). *Proceedings of the 2021 IEEE/CVF Conference on Computer Vision and Pattern Recognition (CVPR2021)*, pp. 10523–10531.

[bb50] Zhang, Y., Chan, S. H. & Lam, E. Y. (2023). *APL Photon.* **8**, 056106.

[bb51] Zhang, Z., Liu, Q. & Wang, Y. (2018). *IEEE Geosci. Remote Sens. Lett.* **15**, 749–753.

