# Supplementary material for: Phase and absorbance retrieval in X-ray holographic microscopy under weak illumination using physics-driven neural networks
Source: J Synchrotron Radiat. 2026 Apr 10;33(Pt 3):794–805. doi: 10.1107/S1600577526003188 (PMC13148600; doi:10.1107/S1600577526003188)
Supplement: Supplementary file 1 [file s-33-00794-sup1.pdf]

JOURNAL OF SYNCHROTRON RADIATION

Volume 33 (2026)

Supporting information for article:

Phase and absorbance retrieval in X-ray holographic microscopy under weak illumination using physics-driven neural networks

Jihwan Kim, Jun Lim, Sugeun Jo, Sungho Park and Sang Joon Lee

Supporting information

Phase and absorbance retrieval in X-ray holographic microscopy under weak illumination using physics-driven neural networks

Jihwan Kim[1], Jun Lim[2], Sugeun Jo[2], Sungho Park[3], Sang Joon Lee[1*]

[1]Department of Mechanical Engineering, Pohang University of Science and Technology, Pohang, Republic of Korea

[2]Pohang Accelerator Laboratory, Pohang University of Science and Technology, Pohang, Republic of Korea

[3]Department of Radiology, Children's Hospital Colorado, University of Colorado Anschutz Medical Campus, Aurora, CO, USA

*Corresponding Author: **Prof. Sang Joon Lee**

Department of Mechanical Engineering, Pohang University of Science and Technology (POSTECH), San 31, Hyoja-dong, Pohang, 37637, Republic of Korea

E-mail: sjlee@postech.ac.kr

Phone: +82-54-279-2169

Fax: +82-54-279-3199

Supplementary Method S1. Segmentation.

Segmentation was performed by using ImageJ, an open-source image-processing software package. In general, thresholding or edge detection methods have been utilized to obtain segmentation masks of target objects. However, the in-focus holograms reconstructed from shot-noise-limited holograms suffered from severe twin-image artifacts induced by shot noise. Therefore, it was difficult to recover clear 2D shape information by using standard segmentation routines. As a result, the segmentation based solely on the in-focus hologram was typically limited to a rough, edge-based estimate of its object mask.

By examining holograms reconstructed at multiple depths near the object plane, we found that the twin image artifacts were temporarily suppressed at certain depths, revealing partial edge structures. The segmentation mask can be obtained by aggregating the edge structures inferred from multiple reconstructed holograms. In addition, it is a usual practice to acquire scanning electron microscope images of target objects prior to X-ray imaging experiment. If the 2D morphology of a target object is known in advance, this information can serve as an auxiliary reference to guide and improve the segmentation process.

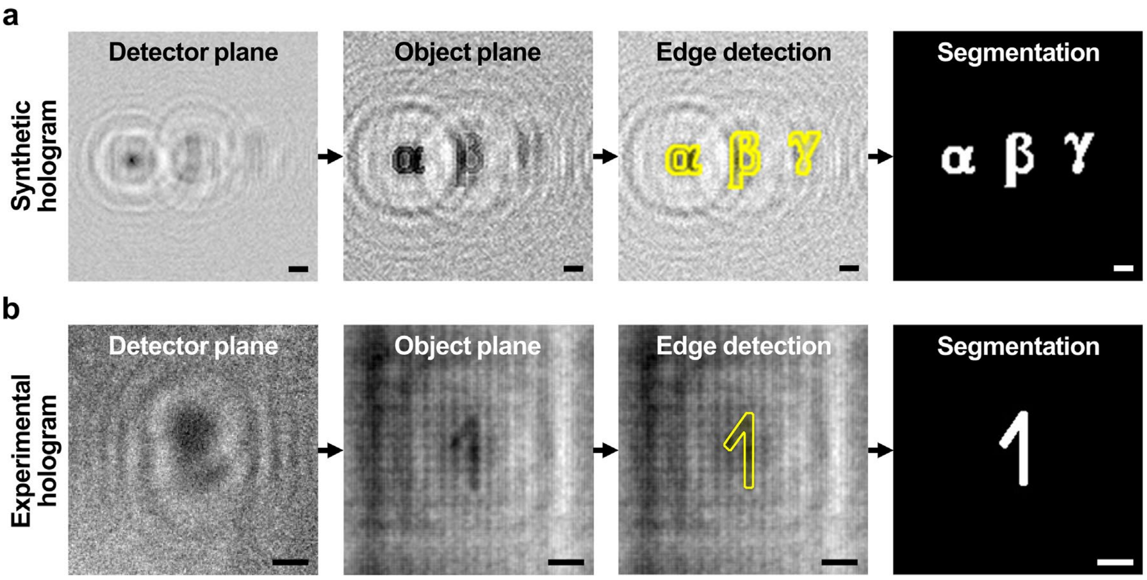

Supplementary Figure S1. Segmentation of (a) synthetic and (b) experimental holograms. Each hologram is back-propagated from the detector plane to the object plane for obtaining its in-focus image. Thresholding and edge-detection methods are applied to the in-focus images with the aid of ImageJ to generate segmentation masks of the target objects. Scale bars: (a) 100 nm; (b) 1000 nm.

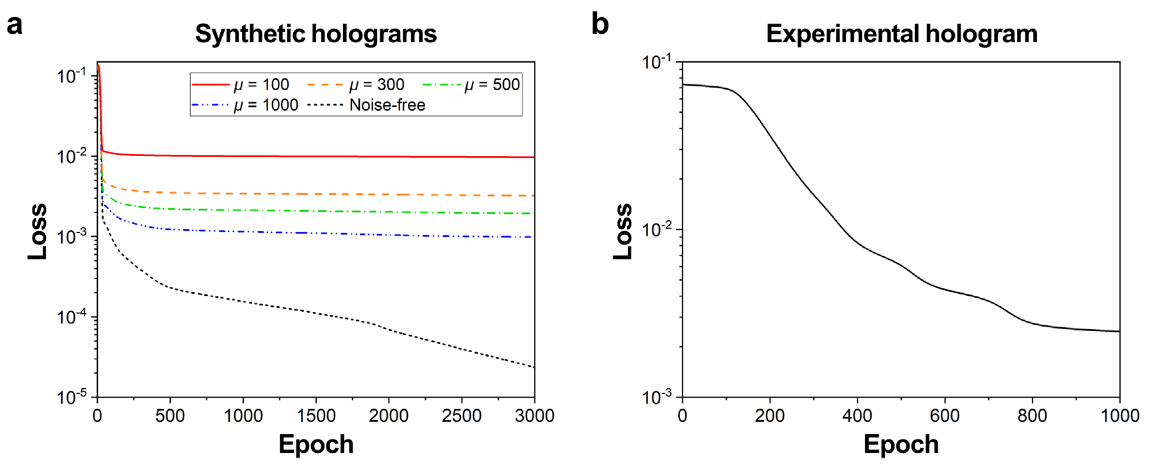

Supplementary Figure S2. Loss curves of MorpHoloNet-X models trained by (a) synthetic and (b) experimental holograms.

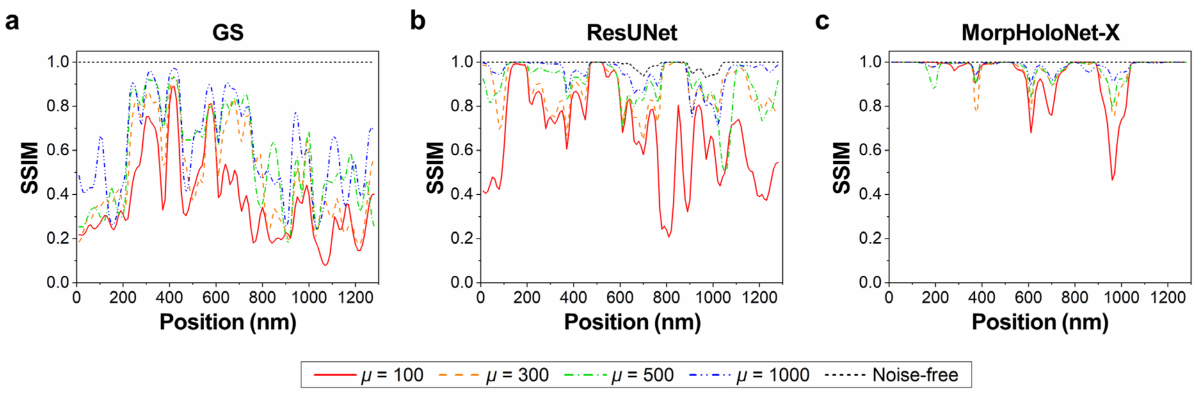

Supplementary Figure S3. Comparisons of structural similarity index measure (SSIM) profiles extracted along the horizontal centerline of the SSIM maps shown in Fig. 3, including phase maps reconstructed from synthetic holograms using (a) the Gerchberg-Saxton (GS) algorithm, (b) residual U-Net (ResUNet), and (c) MorpHoloNet-X.

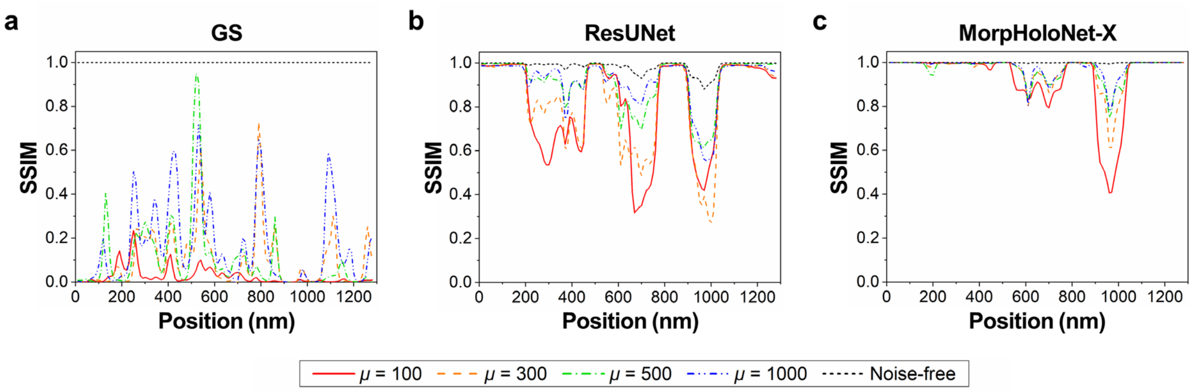

Supplementary Figure S4. Comparisons of structural similarity index measure (SSIM) profiles extracted along the horizontal centerline of the SSIM maps shown in Fig. 5, including absorbance maps reconstructed from synthetic holograms using (a) the Gerchberg-Saxton (GS) algorithm, (b) residual U-Net (ResUNet), and (c) MorpHoloNet-X.
